# Intracellular Trafficking and Persistence of Acinetobacter baumannii Requires Transcription Factor EB

**DOI:** 10.1128/mSphere.00106-18

**Published:** 2018-03-28

**Authors:** Raquel Parra-Millán, David Guerrero-Gómez, Rafael Ayerbe-Algaba, Maria Eugenia Pachón-Ibáñez, Antonio Miranda-Vizuete, Jerónimo Pachón, Younes Smani

**Affiliations:** a Clinic Unit of Infectious Diseases, Microbiology and Preventive Medicine, Institute of Biomedicine of Seville, IBiS, University Hospital Virgen del Rocío/CSIC/University of Seville, Seville, Spain; b Redox Homeostasis Group, Institute of Biomedicine of Seville, IBiS, University Hospital Virgen del Rocío/CSIC/University of Seville, Seville, Spain; University of Kentucky

**Keywords:** *Acinetobacter baumannii*, *Caenorhabditis elegans*, HLH-30, TFEB, bacterial invasion

## Abstract

Acinetobacter baumannii is a significant human pathogen associated with hospital-acquired infections. While adhesion, an initial and important step in A. baumannii infection, is well characterized, the intracellular trafficking of this pathogen inside host cells remains poorly studied. Here, we demonstrate that transcription factor EB (TFEB) is activated after A. baumannii infection of human lung epithelial cells (A549). We also show that TFEB is required for the invasion and persistence inside A549 cells. Consequently, lysosomal biogenesis and autophagy activation were observed after TFEB activation which could increase the death of A549 cells. In addition, using the Caenorhabditis elegans infection model by A. baumannii, the TFEB orthologue HLH-30 was required for survival of the nematode to infection, although nuclear translocation of HLH-30 was not required. These results identify TFEB as a conserved key factor in the pathogenesis of A. baumannii.

**IMPORTANCE** Adhesion is an initial and important step in Acinetobacter baumannii infections. However, the mechanism of entrance and persistence inside host cells is unclear and remains to be understood. In this study, we report that, in addition to its known role in host defense against Gram-positive bacterial infection, TFEB also plays an important role in the intracellular trafficking of A. baumannii in host cells. TFEB was activated shortly after A. baumannii infection and is required for its persistence within host cells. Additionally, using the C. elegans infection model by A. baumannii, the TFEB orthologue HLH-30 was required for survival of the nematode to infection, although nuclear translocation of HLH-30 was not required.

## Abstract (precis)

Adhesion is an initial and important step in Acinetobacter baumannii infections. However, the mechanism of entrance and persistence inside host cells is unclear and remains to be understood. In this study, we report that, in addition to its known role in host defense against Gram-positive bacterial infection, TFEB also plays an important role in the intracellular trafficking of A. baumannii in host cells. TFEB was activated shortly after A. baumannii infection and is required for its persistence within host cells. Additionally, using the C. elegans infection model by A. baumannii, the TFEB orthologue HLH-30 was required for survival of the nematode to infection, although nuclear translocation of HLH-30 was not required.

## INTRODUCTION

Acinetobacter baumannii is an important cause of severe hospital-acquired infections, such as ventilator-associated pneumonia, bacteremia, skin and soft tissue infections, surgical site infections, urinary tract infections, sepsis, and meningitis in humans (1, 2).

The virulence of A. baumannii is based on multiple secreted and surface-associated components. An important group of virulence factors are the outer membrane proteins (OMPs). Among the OMPs, the outer membrane protein A (OmpA) interacts with biotic and abiotic surfaces (3–5). Several other surface and intracellular proteins have been identified, and their role on the virulence of A. baumannii has been characterized (6). For many of these proteins like OmpA, Omp33, phosphorylcholine, lipopolysaccharide, K1 capsular polysaccharide, penicillin-binding protein, and phospholipase D, isogenic mutants are less virulent *in vitro* and *in vivo* (4, 5, 7–12).

Adhesion is an initial and important step in A. baumannii infections. Following adhesion, A. baumannii can invade host cells such as human lung, laryngeal, and cervical epithelial cells (4, 5). A. baumannii enters epithelial cells by way of a microfilament- and microtubule-dependent, zipper-like mechanism, and upon internalization, it localizes to membrane-bound vacuoles (4). Clathrin and β-arrestins are also engaged during the uptake into human lung epithelial cells (8). Interestingly, A. baumannii can persist within host cells; however, no intracellular replication has been reported. *In vitro* and *in vivo* data from our group have demonstrated that A. baumannii induces host cell death and disseminates in tissues and bloodstream (7, 8). Although the dissemination to deeper tissues leads to invasive diseases, their intracellular trafficking is unclear and remains to be understood.

A better understanding of the host factors involved in the A. baumannii intracellular trafficking will help to elucidate the role of this process during infection. Given that A. baumannii is localized to membrane-bound vacuoles (4), we hypothesize that endosomes and lysosomes are involved in the passage of A. baumannii to access the basal side of host cells, through which it can transit across to the underlying tissue. In mammalian host cells, transcription factor EB (TFEB) is known to control the transcription of autophagy and lysosomal biogenesis genes in response to nutritional stress (13). TFEB is regulated by the kinases mammalian target of rapamycin complex 1 (mTORC1) and extracellular signal-regulated kinase 2 (ERK2) (14, 15). During bacterial infection, TFEB and its Caenorhabditis elegans orthologue HLH-30 are regulated by the phospholipase C-protein kinase D (PLC-PKD) pathway (16) and play an important and evolutionary role in the host defense against Gram-positive bacterial infection (16, 17).

In this study, we report that, in addition to its known role in host defense against Gram-positive bacterial infection, TFEB also plays an important role in the intracellular trafficking of a Gram-negative bacillus (GNB), such as A. baumannii, in host cells. TFEB was activated shortly after A. baumannii infection and is required for its persistence within host cells. In addition, HLH-30 is required for C. elegans survival to A. baumannii infection.

## RESULTS

### TFEB expression in A549 cells infected by A. baumannii.

**FIG 1:**
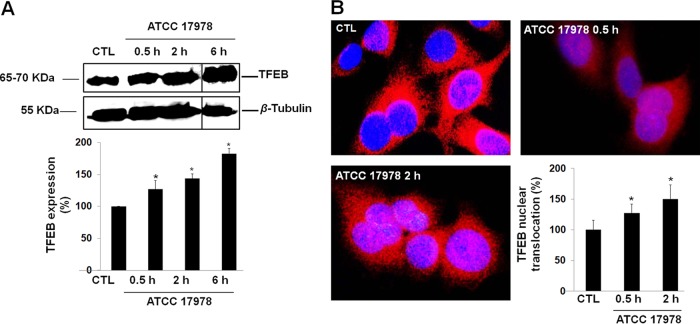
Expression of TFEB in A549 cells by A. baumannii. (A) Western blot analysis of TFEB in A549 cells after incubation with A. baumannii ATCC 17978 for 0.5, 2, and 6 h. The solid black lines in the blots separate the spliced portions of the blots between 2 and 6 h. Values shown in the bar graph are the percentage of TFEB expression in control (CTL) and infected A549 cells. (B) TFEB in A549 cells after incubation with A. baumannii ATCC 17978 for 0.5 and 2 h, immunostaining, and imaging by immunofluorescence microscopy. TFEB detected with rabbit anti-TFEB antibodies and labeled with Alexa Fluor 594-tagged secondary antibodies (red). Blue staining with DAPI shows the location of nuclei of A549 cells. The percentage of TFEB expression in the nuclei of A549 cells was calculated as follows: (number of A549 cells that expressed TFEB in the nuclei of A549 cells/total number of A549 cells) × 100. Results are from three independent experiments, and data are means plus standard errors of the means (SEM) (error bars). Values that are significantly different (*P* < 0.05) between untreated (control [CTL]) and treated groups are indicated by an asterisk.

A. baumannii cellular infection increased the expression of TFEB in a time-dependent manner after 6 h by ≈83% (Fig. 1A). TFEB immunostaining showed that in noninfected control cells, most of TFEB staining is found in the cytoplasm, while 2 h after infection, TFEB is robustly translocated into the nucleus (Fig. 1B).

### Role of TFEB in A. baumannii internalization by A549 cells.

**FIG 2:**
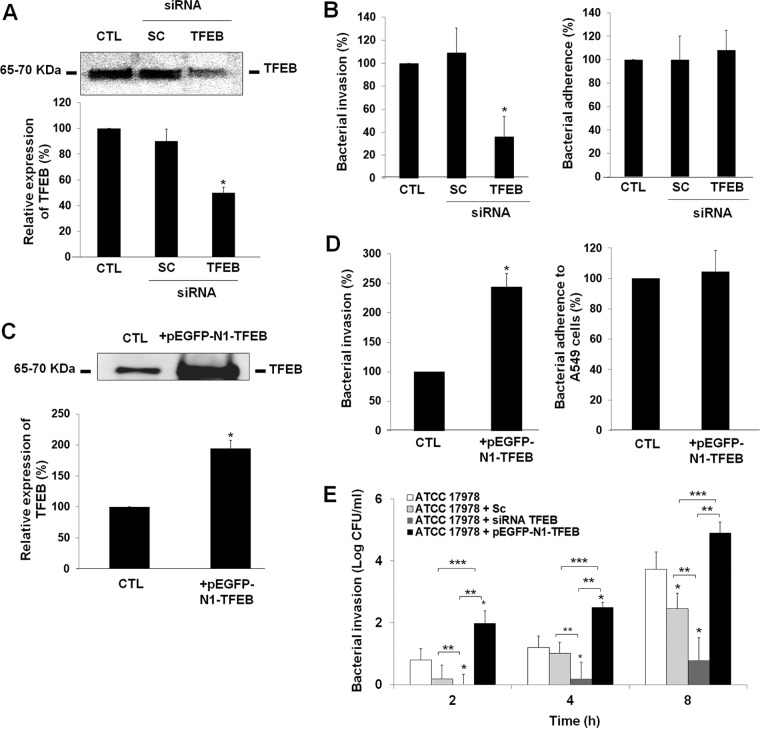
Role of TFEB in A. baumannii internalization by A549 cells. (A and C) Immunoblot analysis of A549 cells transfected with scrambled (SC) and TFEB siRNA and pEGFP-N1-TFEB for 48 and 24 h, respectively. Values in the bar graphs are the percentages of TFEB level in control (CTL) and transfected A549 cells. (B, D, and E) A549 cells were transfected with SC and TFEB siRNA and pEGFP-N1-TFEB and infected with 10^8^ CFU/ml A. baumannii ATCC 17978 for 2, 4, or 8 h. An assay of adherence and invasion of A. baumannii ATCC 17978 into A549 cells was performed as described in Materials and Methods. The effect of TFEB siRNA and pEGFP-N1-TFEB mediated TFEB depletion and overexpression, respectively, on adherence or invasion of A. baumannii ATCC 17978. The percentages of total nontransfected A549 cells and A549 cells incubated with A. baumannii ATCC 17978 are shown for both adhesion and invasion. Results are from three independent experiments, and data are the means plus SEM (error bars). Values for untransfected and transfected groups in panels B and E that are significantly different (*P* < 0.05) are indicated by an asterisk. Values in panel E that are significantly different (*P* < 0.05) are indicated by bars and asterisks as follows: **, ATCC 17978 cells transfected with siRNA TFEB and ATCC 17978 cells or ATCC 17978 cells transfected with pEGFP-N1-TFEB; ***, ATCC 17978 cells and ATCC 17978 cells transfected with pEGFP-N1-TFEB.

We first tested the ability of TFEB small interfering RNA (siRNA) to deplete the TFEB levels in A549 cells. TFEB siRNA transfection reduced the expression of targeted TFEB by ≈55% compared with either nonsilenced or control siRNA-transfected A549 cells (Fig. 2A). TFEB siRNA-transfected A549 cells were found to be effective in decreasing A. baumannii invasion to 36.32% ± 17.6%. However, total cell-adhered bacteria did not differ between control and TFEB siRNA-transfected A549 cells, indicating that invasion did not decrease due to inefficient binding of A. baumannii to A549 cells (Fig. 2B). In contrast, control siRNA-transfected A549 cells did not show significant blocking of A. baumannii adherence and invasion in A549 cells (Fig. 2B). It is important to note that A549 cells transfected with scrambled or TFEB siRNA had no effect on the viability of A549 cells during 24 h of incubation (see Fig. S1 in the supplemental material).

10.1128/mSphere.00106-18.1 FIG S1  Effect of TFEB siRNA on the cellular viability of A549 cells. A549 cells were transfected with siRNA TFEB for 24 h. An assay of A549 cell viability was determined by using the MTT assay. Results are representative of three independent experiments, and values are means plus SEM (error bars). CTL, control; SC, scrambled. Download FIG S1, PDF file, 0.1 MB. Copyright © 2018 Parra-Millán et al. 2018 Parra-Millán et al. This content is distributed under the terms of the Creative Commons Attribution 4.0 International license.

In addition, we evaluated the effect of TFEB overexpression in A549 cells on A. baumannii adherence and invasion. We first tested the ability of pEGFP-N1-TFEB (EGFP stands for enhanced green fluorescent protein) to increase the TFEB levels in A549 cells. pEGFP-N1-TFEB transfection increased the expression of targeted TFEB by ≈80% (Fig. 2C). The pEGFP-N1-TFEB-transfected A549 cells were found to be effective in increasing A. baumannii invasion to 195.69% ± 50.43%. However, total cell-adhered bacteria did not differ between control and pEGFP-N1-TFEB-transfected A549 cells, indicating that this increase was not due to high binding of A. baumannii to A549 cells (Fig. 2D). Interestingly, TFEB siRNA- and pEGFP-N1-TFEB-transfected A549 cells prolonged the significant reduction and increase of the A. baumannii persistence inside A549 cells during 8 h of bacterial infection by 2.95 and 1.17 log CFU/ml compared with control cells, respectively (Fig. 2E). Taken together, these results demonstrate that TFEB is involved in the A. baumannii invasion of human lung epithelial cells.

### Implication of the autophagosome-lysosome system in A. baumannii intracellular trafficking.

**FIG 3:**
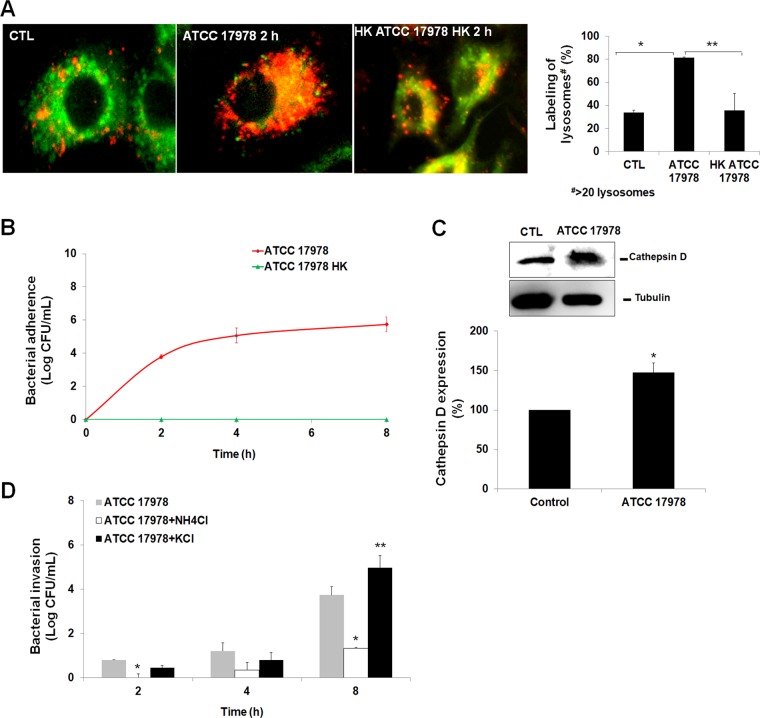
Evaluation of the role of the autophagosome-lysosome system in A. baumannii intracellular trafficking. (A) The lysosomes in A549 cells were incubated with A. baumannii ATCC 17978 for 2 h, immunostained, and imaged by immunofluorescence microscopy. Acidic organelles were detected with LysoTracker red (75 nM), and mitochondria were detected with MitoTracker green (250 nM). The values for labeling of lysosomes in infected A549 cells in the bar graph are percentages compared to the value for noninfected cells. Values that are significantly different (*P* < 0.05) are indicated by bars and asterisks as follows: *, ATCC 17978 and control (CTL) cells; **, ATCC 17978 and heat-killed (HK) ATCC 17978 cells. (B) A. baumannii ATCC 17978 and ATCC 17978 HK invasion into A549 cells for up to 8 h of infection. (C) Western blot analysis of cathepsin D in A549 cells infected with A. baumannii ATCC 17978 for 2 h. Blots were part of the same internally controlled experiment in Fig. 5B. Values are expressed as the percentage of cathepsin D expression level in control and infected A549 cells. Values that are significantly different (*P* < 0.05) are indicated by an asterisk. (D) A. baumannii ATCC 17978 invasion into A549 cells pretreated for 30 min with NH_4_Cl or KCl for various lengths of time up to 8 h of infection. Values that are significantly different (*P* < 0.05) are indicated by asterisks as follows: *, ATCC 17978 cells and ATCC 17978 cells treated with NH_4_Cl; **, ATCC 17978 and ATCC 17978 treated with KCl.

**FIG 4:**
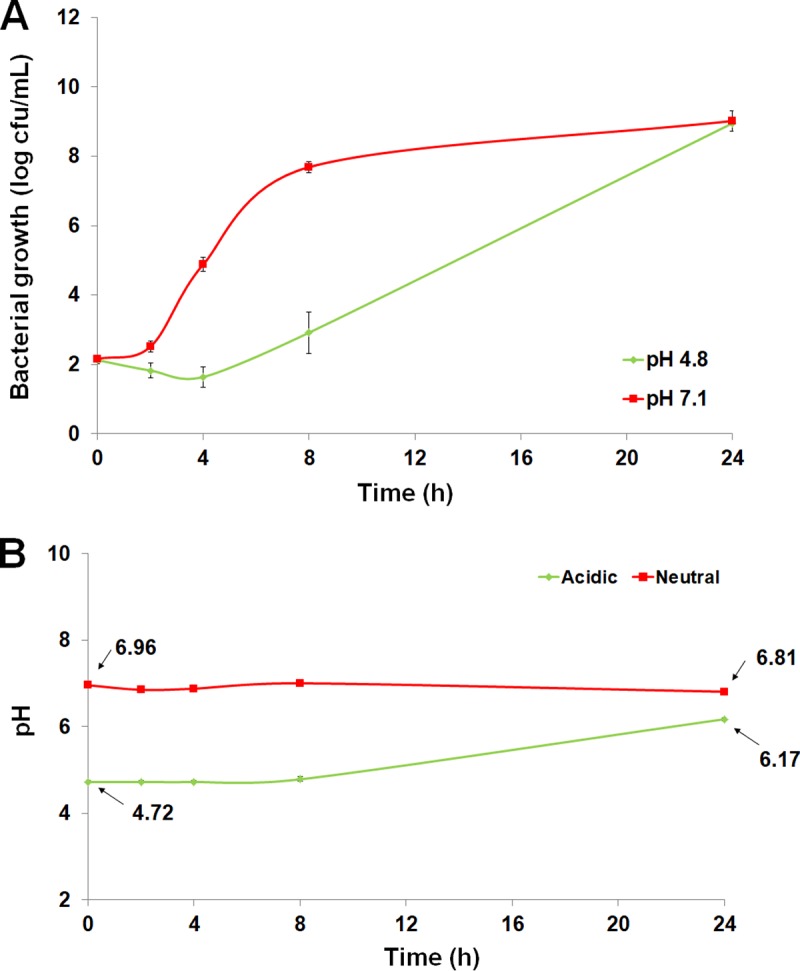
Bacterial acid resistance. (A) Bacterial growth in LB medium during 24 h under acidic or neutral conditions. (B) pH determination during 24 h of LB medium in the presence of A. baumannii ATCC 17978.

To evaluate the role of the autophagosome-lysosome system in A. baumannii intracellular trafficking, we studied the activation of lysosomes upon bacterial infection. We showed that incubation of A549 cells with A. baumannii for 2 h increased the numbers of lysosomes by ≈50%. In contrast, heat-killed A. baumannii did not increase the number of lysosomes significantly (Fig. 3A). In addition, live bacteria persist inside A549 cells for at least 8 h even if the lysosomes were more abundant (Fig. 3B), unlike heat-killed A. baumannii, which activates few lysosomes. Analysis of lysosome membrane damage showed that cathepsin D, an enzyme present inside lysosomes, was released by ≈50% in cytosol after bacterial infection (Fig. 3C). With this precedent in mind and given that lysosomal acidification is crucial to the antimicrobial function of host cells (18), we sought to determine the impact of acidic and neutral conditions on bacterial intracellular viability by testing the effect of NH_4_Cl (19) and KCl (20), respectively, on intracellular persistence of A. baumannii. In A549 cells treated with NH_4_Cl (40 mM, 30 min) before bacterial infection, survival of A. baumannii inside these cells was reduced by 2.42 log CFU/ml (*P* < 0.05) at 8 h (Fig. 3D). In contrast, treatment of A549 cells with KCl (0.2 mM, 30 min) before bacterial infection increased the persistence of A. baumannii inside these cells by 1.24 log CFU/ml (*P* < 0.05) at 8 h (Fig. 3D). It is important to note that treating A549 cells with NH_4_Cl and KCl had no effect on the viability of A549 cells during 24 h of incubation (data not shown), which suggests that persistence of A. baumannii inside A549 cells was not due to the death of these cells. Thus, the observed persistence of A. baumannii inside A549 cells may be due to less lysosome acidification. It is noteworthy that A. baumannii growth under acidic and neutral pH in LB broth at 24 h was unchanged, although their growth dynamic is different in the first hours of bacterial growth (Fig. 4A). In acidic conditions, the pH value of LB medium during A. baumannii growth shifted from 4.72 to 6.17 between 0 and 24 h (Fig. 4B), while the pH of the LB medium after 24 h of A. baumannii growth in neutral pH was unchanged and shifted only from 6.96 to 6.81 (Fig. 4B). These data confirm that A. baumannii is acid resistant, as are other GNB (21), and suggest that in A549 cells, A. baumannii is able to resist lysosome acidification.

**FIG 5:**
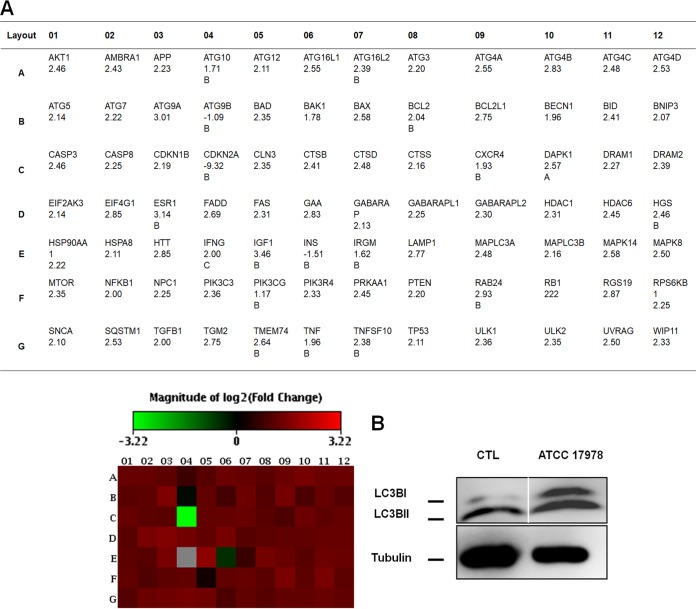
A. baumannii stimulates the autophagy. (A) Expression of human autophagy genes after A. baumannii infection. Total RNA was isolated from A549 cells infected with A. baumannii ATCC 17978 and uninfected cells. cDNAs were synthesized by reverse transcription of the total RNA. A real-time PCR analysis was performed by using the Stratagene Mx3005p system. Samples were normalized to beta-2-microglobulin. Human autophagy gene expression after infection is represented by the heat map. Results are representative of two independent experiments. (B) Western blot analysis of LC3B in A549 cells infected with A. baumannii ATCC 17978 for 2 h. The blots were part of the same internally controlled experiment in Fig. 3C. Results are representative of three independent experiments. The solid white line separates the spliced portions between control and infected cells.

Lu et al. showed that *Streptococcus* group A induces low lysosome acidification without sufficient activation of autophagy in endothelial cells (18). To test whether A. baumannii may activate autophagy, we determined the expression profiles of 84 autophagic genes by real-time PCR in A. baumannii-infected A549 cells compared with noninfected control cells. After 2 h of bacterial infection, 79 genes were upregulated (Fig. 5A), including MAP1LC3B encoding LC3B, the most studied gene in autophagy (22). Western blot analysis of LC3BII showed that LC3BII protein levels were increased by ≈50% (Fig. 5B) and confirmed the result obtained by the autophagic gene expression profiling.

**FIG 6:**
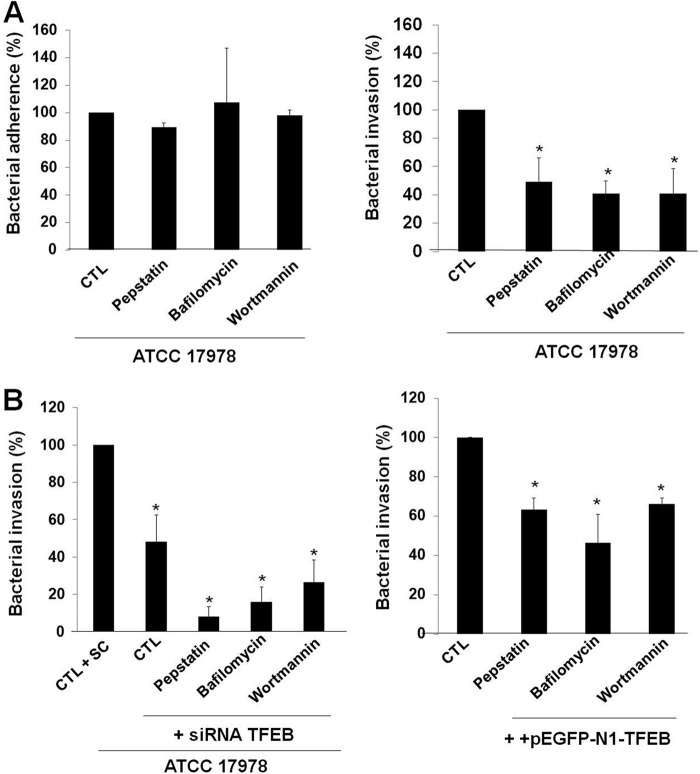
A. baumannii activates the autophagosome-lysosome system. (A and B) Additive effect of autophagy and TFEB on *A. baumannii* internalization by A549 cells. A549 cells were transfected with scrambled (SC) or TFEB siRNA or pEGFP-N1-TFEB and treated with pepstatin (20 µg/ml), bafilomycin (0.8 µM), or wortmannin (1 µM), and infected with 10^8^ CFU/ml *A. baumannii* ATCC 17978 for 2 h to study bacterial adherence and invasion to host cells. Results are representative of three independent experiments, and data are the means plus SEM. Values for treated and untreated groups that are significantly different (*P* < 0.05): are indicated by an asterisk. CTL, control.

Next, we addressed whether the autophagosome-lysosome system might also participate in the intracellular trafficking of A. baumannii inside A549 cells. In this regard, pharmacological inhibition by pepstatin, an inhibitor of lysosomal degradation (23), bafilomycin, an inhibitor of the fusion between autophagosomes and lysosomes (24), or wortmannin, an inhibitor of the class III phosphatidylinositol 3-kinase (PI3K) activity (24), reduced A. baumannii invasion inside A549 cells to 49.14% ± 17.11%, 41.02% ± 8.97%, or 40.79% ± 17.9%, respectively. However, the total numbers of cell-adhered bacteria did not differ in untreated and pepstatin-, bafilomycin-, or wortmannin-treated A549 cells, indicating that the inhibition was not due to inefficient binding of A. baumannii to A549 cells (Fig. 6A). Interestingly, the pretreatment of A549 cells by pepstatin, bafilomycin, or wortmannin together with the inhibition of TFEB by TFEB siRNA amplified the reduction of A. baumannii invasion of A549 cells to 8.25% ± 5.13%, 16.05% ± 7.73%, or 26.6% ± 11.89%, respectively, compared to treatment with pepstatin, bafilomycin, wortmannin, or TFEB siRNA alone (Fig. 6B). Conversely, the pretreatment of A549 cells by pepstatin, bafilomycin, or wortmannin together with the TFEB overexpression by pEGFP-N1-TFEB reduced A. baumannii invasion of A549 cells to 63.34% ± 5.89%, 46.5% ± 14.17%, or 66.29% ± 11.89%, respectively, reducing invasion less than treatment with pepstatin, bafilomycin, or wortmannin alone (Fig. 6B). It is important to note that treating A549 cells with pepstatin, bafilomycin, and wortmannin had no effect on the viability of A549 cells during 24 h of incubation (Fig. S2). Together, these results support a key role for autophagosome-lysosome system in the intracellular persistence of A. baumannii.

10.1128/mSphere.00106-18.2 FIG S2  Effects of inhibitors of the autophagosome-lysosome system on the cellular viability of A549 cells. A549 cells were treated with pepstatin (20 µg/ml), bafilomycin (0.8 µM), or wortmannin (1 µM) for 24 h. An assay of the viability of A549 cells was determined by using the MTT assay. Results are representative of three independent experiments, and values are means plus SEM. CTL, control. Download FIG S2, PDF file, 0.1 MB. Copyright © 2018 Parra-Millán et al. 2018 Parra-Millán et al. This content is distributed under the terms of the Creative Commons Attribution 4.0 International license.

Collectively, these data suggest a hypothetical model whereby infection triggers TFEB activation, which could induce the autophagosome-lysosome system to promote the intracellular trafficking and persistence of A. baumannii within cells.

### HLH-30 is necessary for C. elegans survival, but not for A. baumannii infection.

To address the role of TFEB in the context of an infective process in a complete organism and because TFEB and its C. elegans orthologue HLH-30 are both regulated by infection (16, 17), we hypothesized that HLH-30 might also be required for C. elegans to cope with A. baumannii infection.

**FIG 7:**
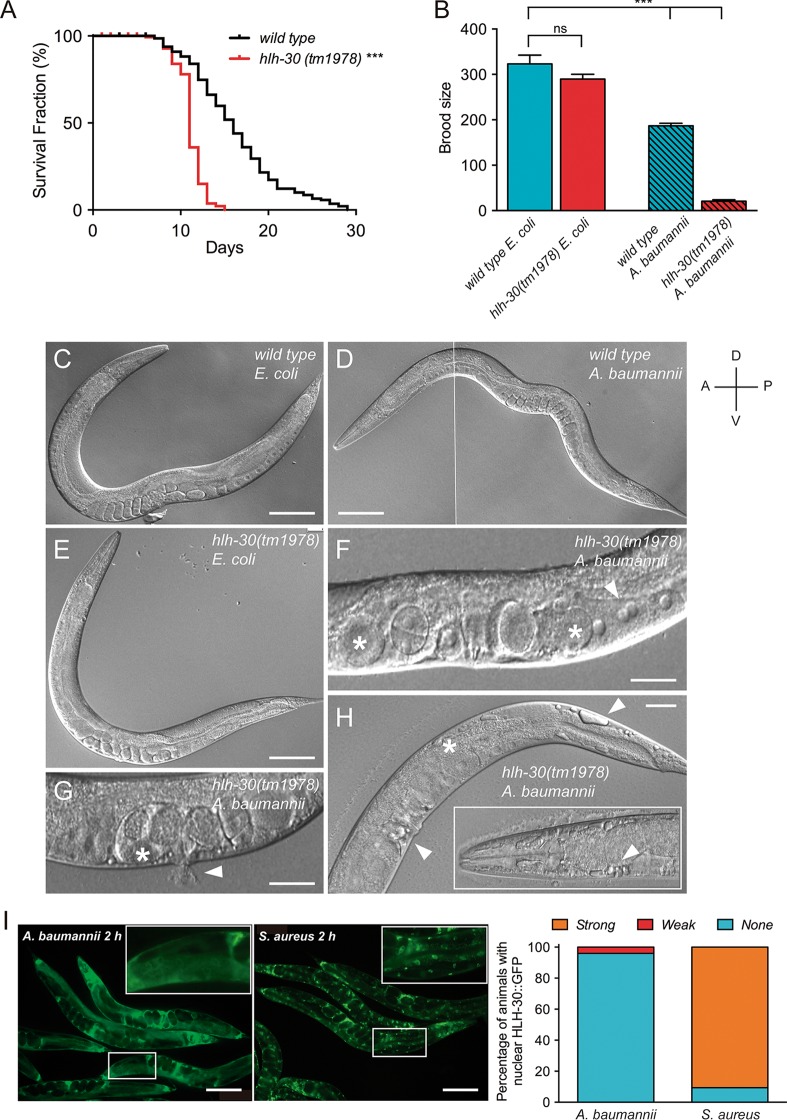
HLH-30 is required for *C*. *elegans* survival against *A*. *baumannii* infection. (A) Longevity assay of *hlh-30*(*tm1978*) mutant worms compared to wild-type control worms growing on A. baumannii at 20°C. Survival of wild-type (*n* = 140) and *hlh-30*(*tm1978*) mutants (*n* = 134) of C. elegans when growing at the same temperature in the nonpathogenic E. coli OP50 or in the pathogenic A. baumannii ATCC 17978. Two independent experiments were performed, and the data for both experiments are shown (***, *P* < 0.001). (B) Brood size quantification of wild-type and *hlh-30*(*tm1978*) mutants growing from eggs on E. coli OP50 and A. baumannii. Values are means plus SEM for 30 individuals (***, *P* < 0.001; ns, not significantly different). (C to H) Differential interference contrast (DIC) micrographs of wild-type and *hlh-30*(*tm1978*) mutants growing from eggs on E. coli OP50 and A. baumannii showing alterations in worm germline like enlarged oocytes and embryos (white asterisks in panels F, G, and H), binucleated oocytes (white arrowhead in panel F), vulva extrusion (white arrowhead in panel G), and extensive blebbing in the intestine, vulva, and head regions (H). The solid white line in panel D separates the spliced portions. (I) Fluorescence micrographs (left) and quantification (right) of HLH-30::GFP nuclear translocation in intestinal cells of transgenic worms expressing the integrated array *sqIs17 [Phlh-30*::*hlh-30*::*GFP*;* rol-6*(*su1006*)] when grown in S. aureus 29213 or A. baumannii ATCC 17978. Bars, 100 µm (C, D, E, and I) and 50 µm (F, G, and H). D, dorsal; V, ventral; A, anterior; P, posterior.

To test this idea, we first performed a longevity assay of wild-type and *hlh-30*(*tm1978*) mutant worms growing in A. baumannii culture. Interestingly, *hlh-30* mutant worms have a strong reduction in the mean life span compared with wild-type control worms (11 versus 16 days). In addition, a dramatic decrease occurred in the maximum life span of *hlh-30* mutants compared to the wild-type controls (29 versus 15 days) (Fig. 7A). Note that the life span of *hlh-30* mutant worms was barely affected when grown on Escherichia coli OP50 (17). Similarly, while the brood size of *hlh-30* mutant worms was not different from that of wild-type controls when grown on E. coli OP50, the brood sizes of both wild-type and *hlh-30* mutant worms were affected significantly when raised on A. baumannii, although to different extents. Thus, wild-type control worms had a brood size reduction of 42%, while *hlh-30* mutant worms displayed a dramatic reduction of 93% when grown in A. baumannii compared to E. coli OP50 (Fig. 7B), suggesting that A. baumannii causes defects in germline function in C. elegans. Visual inspection by differential interference contrast (DIC) microscopy of *hlh-30* mutant and wild-type worms growing on A. baumannii confirmed this hypothesis and identified severe germline phenotypes like oocytes with abnormal size (enlarged and small), binucleated oocytes, enlarged and deformed embryos, blisters and blebs in the head, vulva, and tail and also in some cases extruded intestinal/uterine contents (Fig. 7C to H). Together, these data suggest that HLH-30 is a key factor for C. elegans to survive A. baumannii infection.

A fluorescent HLH-30::GFP reporter was previously shown to translocate to the nucleus upon Staphylococcus aureus infection (16, 17). We then tested whether A. baumannii infection would induce a similar response. Unexpectedly, and in sharp contrast to S. aureus infection, A. baumannii induced very weak HLH-30 nuclear translocation in C. elegans intestinal cells after 2 h of infection (Fig. 7I) or 12 to 24 h (data not shown).

## DISCUSSION

The present study provides new data highlighting the nature of the mechanism involved in the intracellular trafficking and persistence of A. baumannii into human and C. elegans host cells. Here, we provide the first evidence of an essential role played by TFEB/HLH-30 in entrance and persistence of A. baumannii into human host cells and in the longevity and germline function of C. elegans infected with A. baumannii.

This study showed that TFEB is one of the intracellular factors involved in the invasion of epithelial cells by A. baumannii, consistent with other factors such as clathrin and β-arrestins reported for A. baumannii (8) and other pathogens (25, 26). Previous independent work showed that lipopolysaccharide can stimulate TFEB in murine dendritic cells (27), and activation of TFEB was shown to be important for host defense against staphylococcal pore-forming toxins and S. aureus (17, 28). However, to date, the question is how is TFEB relevant to the persistence of A. baumannii inside infected host cells.

Here, we showed that TFEB is overexpressed in infected human lung epithelial cells. Recently, a hypothetical model has been established in macrophages in which Salmonella enterica infection activates Gαq, presumably via an unidentified G-protein-coupled receptor Gαq, which in turn activates PLC. PLC generates diacylglycerol (DAG), resulting in the activation of PKD (16). PKD activation is required for TFEB nuclear translocation (16). Ca^2+^ is an important component in the activation of TFEB after E. coli infection (29). Evidence supports this hypothetical model in which we demonstrated previously that A. baumannii expressing phosphorylcholine, an OMP decorated by phospholipid, adheres to human lung epithelial cells via platelet-activating factor receptor, which thereafter activates a cascade of pathways composed of G-protein-coupled PLC, clathrin, and β-arrestins, which are required for invasion of A. baumannii into human lung epithelial cells (8).

Activation of TFEB is also important for lysosome biogenesis (13). We observed that A. baumannii, which induced TFEB expression, increases the number of lysosomes, which have been implicated in bacterial lysis (30). However, A. baumannii is still present at higher levels inside host cells during 8 h. This phenomenon occurs with other intracellular pathogens in which lysosomes lose their activities against these pathogens (30–33). A possible explanation could be the destabilization of their membrane, as we revealed in this study by the presence of cathepsin D outside the lysosomes. These results are consistent with previous observations that cathepsin D is released into the cytosol after lysosome destabilization in different types of cells (34, 35). Another explanation would be the shift of the lysosomes from acid to neutral conditions. By analyzing the growth conditions of A. baumannii in broth and in host cells, we show that this pathogen is not acidophilic and required neutral conditions to replicate. The persistence of A. baumannii inside host cells could explain the loss of acidic conditions inside lysosomes. Similar data were observed in other studies with other pathogens in which insufficient acidification of phagosomal environment facilitates *Streptococcus pyogenes*, Serratia marcescens, and *Candida glabrata* survival and growth in host cells (36–38).

It is noteworthy that TFEB response and effect were different following the type of bacterial stimulus. Different responses have been observed in Gram-positive (S. aureus) and Gram-negative (S. enterica) bacteria and in Mycobacterium tuberculosis (16, 17, 39). In this study, we demonstrate the role of TFEB in the persistence of A. baumannii inside host cells. S. enterica, another GNB, increases activation and translocation of TFEB into the nuclei of macrophages (16). Moreover, Visvikis et al. showed that TFEB, after stimulus by S. aureus, was required for proper transcription and induction of several proinflammatory cytokines and chemokines (17). In another group of bacteria, M. tuberculosis through miR33 is able to persist inside host by the repression of TFEB and expression of autophagic genes (39), and therefore low lysosome formation.

To study the role of TFEB in A. baumannii infection in a complete organism, we moved to the invertebrate model C. elegans. The worm TFEB orthologue HLH-30 is important for C. elegans survival against A. baumannii, as observed for other pathogens such as S. aureus (17), as the absence of *hlh-30* causes a dramatic decrease in worm life span and brood size, with the latter a consequence of severe deleterious phenotypes affecting the germline. While S. aureus induces rapid and robust nuclear translocation of HLH-30 (17), A. baumannii induces very weak HLH-30 nuclear translocation in intestinal cells of C. elegans. One possible explanation is that the nuclear translocation dynamics of the HLH-30::GFP reporter is different in Gram-positive versus Gram-negative pathogens even if HLH-30 is still needed for survival against both. Another possibility is that a small, undetected fraction of HLH-30 is still translocated into the nucleus upon A. baumannii exposure, likely masked by the stronger diffuse cytoplasmic labeling, sufficient to trigger the HLH-30-dependent transcriptional program necessary to combat the infection.

In summary, we have demonstrated that TFEB is required for the invasion and persistence of A. baumannii inside human eukaryotic cells. Additionally, using the C. elegans infection model by A. baumannii, we have showed that the TFEB orthologue HLH-30 was required for survival of the nematode to infection, although HLH-30 translocation in the nucleus of C. elegans is very weak.

## MATERIALS AND METHODS

### Bacterial and C. elegans strains, plasmids, and growth conditions.

A. baumannii ATCC 17978 (alive and heat killed [HK]), E. coli OP50, and S. aureus ATCC 29213 were used in this study. A. baumannii was grown in LB medium (Sigma, Spain) at 37°C for 20 to 24 h, washed with phosphate-buffered saline (PBS), and resuspended in Dulbecco modified Eagle medium (DMEM) prior to use in eukaryotic cell culture experiments.

For C. elegans experiments, A. baumannii and S. aureus were grown overnight at 37°C in LB medium. The stationary culture was then diluted to an optical density at 600 nm (OD_600_) of 0.7, and 100-µl portions were seeded onto 60-mm nematode growth medium (NGM) plates (42). Plates were incubated at 37°C for 24 h before use. When using E. coli OP50, 100-µl portions of overnight culture were seeded onto 60-mm NGM plates and incubated for 48 h before use.

The C. elegans strains used in this work were N2 (wild type), VT1584 [*hlh-30*(*tm1978*) IV] (40), and MAH240 {*sqIs17* [*Phlh-30*::*hlh-30*::*GFP; rol-6*(*su1006*)]} (36). Some of these strains were provided by the CGC, which is funded by the NIH Office of Research Infrastructure Programs (P40 OD010440).

Plasmid pEGFP-N1-TFEB was a gift from Shawn Ferguson (Addgene plasmid 38119, Addgene USA) (31).

### Human cell culture.

Type II pneumocyte cell line A549 derived from a human lung carcinoma (ATCC CCL-185) was grown in DMEM supplemented with 10% heat-inactivated fetal bovine serum (FBS), vancomycin (50 μg/ml), gentamicin (20 μg/ml), amphotericin B (0.25 μg/ml) (Invitrogen, Spain), and 1% HEPES in a humidified incubator with 5% CO_2_ at 37°C. A549 cells were routinely passaged every 3 or 4 days. Immediately before infection, A549 cells were washed three times with prewarmed PBS and further incubated in DMEM without FBS and antibiotics.

### siRNA transfection.

Chemically synthesized, double-stranded small interfering RNAs (siRNAs) for TFEB and control were purchased from Qiagen. The siRNA sequences targeting TFEB and control are 5′-CTCGACGTTCTGCAAGGTCTA-3′ and 5′-AATTCTCCGAACGTGTCACGT-3′, respectively. Forty to 50% confluent A549 cells (1 × 10^6^ cells/well) in the wells on a six-well plate, split at least 24 h before transfection, were transfected using the HiPerfect transfection reagent (Qiagen, Spain) according to a modified version of the manufacturer’s instructions. Briefly, 6 µl of HiPerfect transfection reagent was added to 100-µl mixtures of siRNAs (10 nM) and Optimem (Invitrogen, Spain), mixed by inversion, and incubated for 15 min at room temperature. The entire transfection mixture was added to A549 cells containing fresh serum-free Optimem. After A549 cells were incubated for 4 h at 37°C, an additional 3 ml of DMEM supplemented with FBS and antibiotics was added to each well on the plate. After additional incubation for 48 h, TFEB expression was studied by Western blotting.

### Plasmid transfection.

Forty to 50% confluent A549 cells (1 × 10^6^ cells/well) in the wells of a six-well plate, split at least 24 h before transfection, were transfected using the Lipofectamine transfection reagent (Invitrogen, Spain). Briefly, 12 µl of Lipofectamine transfection reagent was added to 150 µl of Optimem, and 3 µg of pEGFP-N1-TFEB was added to another 150 µl of Optimem, mixed by inversion, and incubated for 5 min at room temperature. Both mixtures were then mixed and incubated for 25 min at room temperature. The entire transfection mixture was added to A549 cells containing fresh serum-free Optimem. After A549 cells were incubated for 4 h at 37°C, an additional 3 ml of DMEM supplemented with FBS and antibiotics was added to the wells. After additional incubation for 24 h, TFEB expression was studied by Western blotting.

### Adhesion and internalization assays.

Control and transfected A549 cells (transfected with scrambled and TFEB siRNA or pEGFP-N1-TFEB) were infected with 1 × 10^8^ CFU/ml of A. baumannii ATCC 17978 (live or HK) at a multiplicity of infection (MOI) of 100 for 2, 4, and 8 h with 5% CO_2_ at 37°C. Subsequently, infected A549 cells were washed five times with prewarmed PBS and lysed with 0.5% Triton X-100. Diluted lysates were plated onto blood agar (Columbia blood agar; Becton Dickinson Microbiology Systems, USA) and incubated at 37°C for 24 h for enumeration of developed colonies and then the determination of the number of bacteria that attached to A549 cells. Alternatively, to determine the number of internalized bacteria, the wells were washed and incubated a further 30 min in DMEM supplemented with 256 µg/ml gentamicin to kill extracellular bacteria (the ATCC 17978 gentamicin MIC is 0.5 mg/liter) and then washed three times with PBS to remove antibiotic. The number of internalized bacteria was determined as described above.

Moreover, control and transfected A549 cells (transfected with scrambled and TFEB siRNA or pEGFP-N1-TFEB) were pretreated with pepstatin (20 µg/ml, 30 min), bafilomycin (0.8 µM, 30 min), wortmannin (1 µM, 30 min), NH_4_Cl (40 mM, 30 min) or KCl (0.2 mM, 30 min); and infected with 1. 10^8^ CFU/ml of A. baumannii ATCC 17978 for 2 h with 5% CO_2_ at 37°C. Subsequently, adhesion and internalization assays were performed as mentioned previously.

### Cellular viability.

Control A549 cells were transfected with TFEB siRNA or treated with pepstatin (20 µg/ml), bafilomycin (0.8 µM), or wortmannin (1 µM) for 24 h with 5% CO_2_ at 37°C. Prior to the evaluation of cellular viability, we first washed A549 cells three times with prewarmed PBS and then quantitatively assessed cellular viability by monitoring the mitochondrial reduction activity by the 3-(4,5-dimethylthiazol-2-yl)-2,5-diphenyltetrazolium bromide (MTT) assay described previously (8). The percentage of cellular viability was calculated from the optical density (OD) as follows: (OD of treated cells/mean OD of nontreated cells) × 100.

### Immunofluorescence.

The A549 cells (1 × 10^5^ cells) plated on coverslips, infected with A. baumannii ATCC 17978 (MOI of 100) or not infected with A. baumannii at 37°C for 2 h were removed and washed five times with cold PBS. The A549 cells on the coverslips were fixed in methanol for 8 min at −20°C, permeabilized with 0.5% Triton X-100, and blocked with 20% pork serum in PBS. The primary antibody, rabbit anti-human TFEB (Cell Signaling Technology, USA), was used at a dilution of 1:100 in PBS containing 1% bovine serum albumin (BSA) for 2 h. After the coverslips were washed with PBS, they were incubated with the secondary antibody, Alexa Fluor 594-conjugated goat anti-rabbit IgG (Invitrogen, Spain) at a dilution of 1:200 in PBS containing 1% BSA for 1 h. The fixed coverslips were incubated for 10 min at room temperature with 4′,6′-diamidino-2-phenylindole (DAPI) (AppliChem, Germany) (0.5 μg/ml), washed with PBS, mounted in fluorescence mounting medium (Dako Cytomation, Spain), and visualized using a Leica fluorescence microscope (DM-6000; Leica Microsystems Wetzlar GmbH, Germany).

### Lysosome staining.

Detection of lysosomes in A549 cells after incubation with A. baumannii ATCC 17978 (live and HK) for 2 h was performed and imaged by immunofluorescence microscopy. Control and infected (MOI of 100) A549 cells (1 × 10^6^ cells/well) were incubated with 75 nM LysoTracker red (Invitrogen, Spain) for 90 min and with 250 nM MitoTracker green (Invitrogen, Spain), a marker of mitochondria, for 45 min. Subsequently, lysosomes and mitochondria were visualized using a Leica fluorescence microscope (DM-6000; Leica Microsystems Wetzlar GmbH, Germany).

### Western blot immunoblotting.

Control and transfected A549 cells (transfected with TFEB siRNA or pEGFP-N1-TFEB) (1 × 10^6^ cells) were collected, homogenized in radioimmunoprecipitation assay (RIPA) buffer (Sigma, Spain) supplemented with 1 mM phenylmethylsulfonyl fluoride (PMSF) and 10% cocktail of protease inhibitors (Sigma, Spain), and centrifuged at 13,000 × *g* for 20 min at 4°C. The amount of proteins was determined using the bicinchoninic acid (BCA) assay, and the Western blot protocol was performed as described previously (41). The specific modifications were that 10% SDS-PAGE was used for TFEB and cathepsin expression analysis and 4 to 15% mini-PROTEAN TGX gels (Bio-Rad, Spain) were used for LC3B expression analysis. The primary antibodies used were rabbit anti-human TFEB (Cell Signaling Technology, USA), rabbit anti-human cathepsin D (Cell Signalling Technology, USA), rabbit anti-human LC3B (Cell Signalling Technology, USA), and rabbit β-tubulin (Cell Signaling Technology, USA) (at dilutions of 1:1,000, 1:1,000, 1:500, and 1:1,000, respectively), and the secondary antibody used was horseradish peroxidase-conjugated donkey anti-rabbit IgG (GE Healthcare, UK) (at dilutions of 1:5,000, 1:5,000, 1:1,000, and 1:2,000, respectively). The amounts of cathepsin D and LC3BII proteins in A549 cells for each treatment were normalized to amounts in the control samples and divided by the corresponding value of β-tubulin, which was also normalized to control samples.

### Human autophagy gene expression.

The human autophagy RT^2^ Profiler PCR array (Qiagen, Germany) was used to study the expression profiles of 84 autophagy-specific genes in accordance with the manufacturer’s recommendations. Briefly, total RNA was isolated from A549 cells (1 × 10^6^ CFU/well) infected by A. baumannii ATCC 17978 (MOI of 100) for 2 h, and noninfected A549 (control cells) following the specifications of the miRNeasy minikit (Qiagen, Spain). cDNAs were synthesized by reverse transcription from 0.5 μg of total RNA using the RT^2^ First Strand kit (Qiagen, Germany). Then, cDNA was added to the reaction mixture containing RT^2^ SYBR green quantitative PCR (qPCR) mastermix (Qiagen, Germany) and aliquoted across the human autophagy 96-well RT^2^ Profiler PCR array. DNA was amplified using a Stratagene Mx3005p system as follows: (i) 10 min at 95°C and (ii) 40 cycles, with 1 cycle consisting of 15 s at 95°C and 1 min at 60°C. Relative expression of genes was determined using the 2^−ΔΔCt^ within the control group and tested group for each gene. Samples were normalized to beta-2-microglobulin. Human autophagy gene expression after infection was represented by a heat map. Two independent experiments were performed.

### Bacterial growth at different pHs.

To obtain growth curves, A. baumannii ATCC 17978 was grown on LB medium adjusted to pH 4.8 or 7.1 in triplicate. Bacterial cultures were incubated at 37°C, and viable counts were determined by serial dilution at 0, 2, 4, 8, and 24 h by plating 100-μl portions of test cultures or dilutions at the indicated times onto sheep blood agar plates (Becton Dickinson, USA). The plates were incubated for 24 h, and after colony counts, the log_10_ of viable cells (CFU/milliliter) was determined.

### C. elegans life span, brood size, phenotypic analysis. and HLH-30::GFP nuclear translocation assays.

For life span experiments, gravid hermaphrodites were bleached with a solution containing 5 N NaOH and 1% NaOCl. The resulting eggs were washed with M9 buffer and transferred onto NGM plates seeded with fresh A. baumannii. Age-synchronized eggs were grown at 20°C until animals reached larval stage 4 (L4). The L4 worms were then transferred to fresh plates seeded with A. baumannii in groups of 30 worms per plate for a total of 150 individuals per experiment. The day animals reached L4 was used at time zero. The animals were maintained at 20°C, transferred to fresh plates every day until the end of the reproductive period, and then shifted every 2 or 3 days but monitored daily for dead animals. Worms that did not respond to stimulation by touch and displayed no pharyngeal pumping were scored as dead. The survival of the *hlh-30*(*tm1978*) animals was compared to the survival of wild-type controls using the log rank (Mantel-Cox) test.

For brood size experiments, wild-type and *hlh-30*(*tm1978*) synchronized L1 larvae were grown at 20°C in E. coli OP50 or A. baumannii plates until the worms reached L4. Then, 30 L4 animals were transferred to new plates seeded with fresh E. coli OP50 or A. baumannii and maintained at 20°C. Worms were allowed to lay eggs and transferred to fresh plates every 2 days until the end of the reproductive period. Live progeny was counted 48 h after the eggs were laid.

To document morphological phenotypes, synchronized wild-type and *hlh-30*(*tm1978*) L1 animals were grown on NGM plates seeded with *E*. *coli* OP50 or A. baumannii at 20°C until worms reached young adult stage. Worms were mounted on a glass slide with agarose pad and immobilized with sodium azide (5%). Phenotypes were observed with an Olympus BX61 microscope using differential interference contrast optics.

HLH-30::GFP nuclear translocation quantification was performed for first day adult worms grown at 20°C in NGM plates seeded with OP50 and subsequently exposed for 2 h to A. baumannii or S. aureus at 20°C. The animals were then mounted on glass slides with agarose pads and paralyzed with sodium azide (5%). HLH-30::GFP subcellular localization was visualized using an Olympus BX61 fluorescence microscope.

### Statistical analysis.

For *in vitro* and *in vivo* studies, continuous group data are means ± standard errors of the means (SEM). Student’s *t* test was used to determine differences between means. For life span experiments, survival of the mutant and wild-type animals were compared using the log rank test (Mantel-Cox). Differences were considered significant at *P* < 0.05. The SPSS (version 17.0) statistical package was used (SPSS Inc.).
