# Supplementary figures and images for: Intracellular Trafficking and Persistence of Acinetobacter baumannii Requires Transcription Factor EB
Source: mSphere. 2018 Mar 28;3(2):e00106-18. doi: 10.1128/mSphere.00106-18 (PMC5874439; doi:10.1128/mSphere.00106-18)

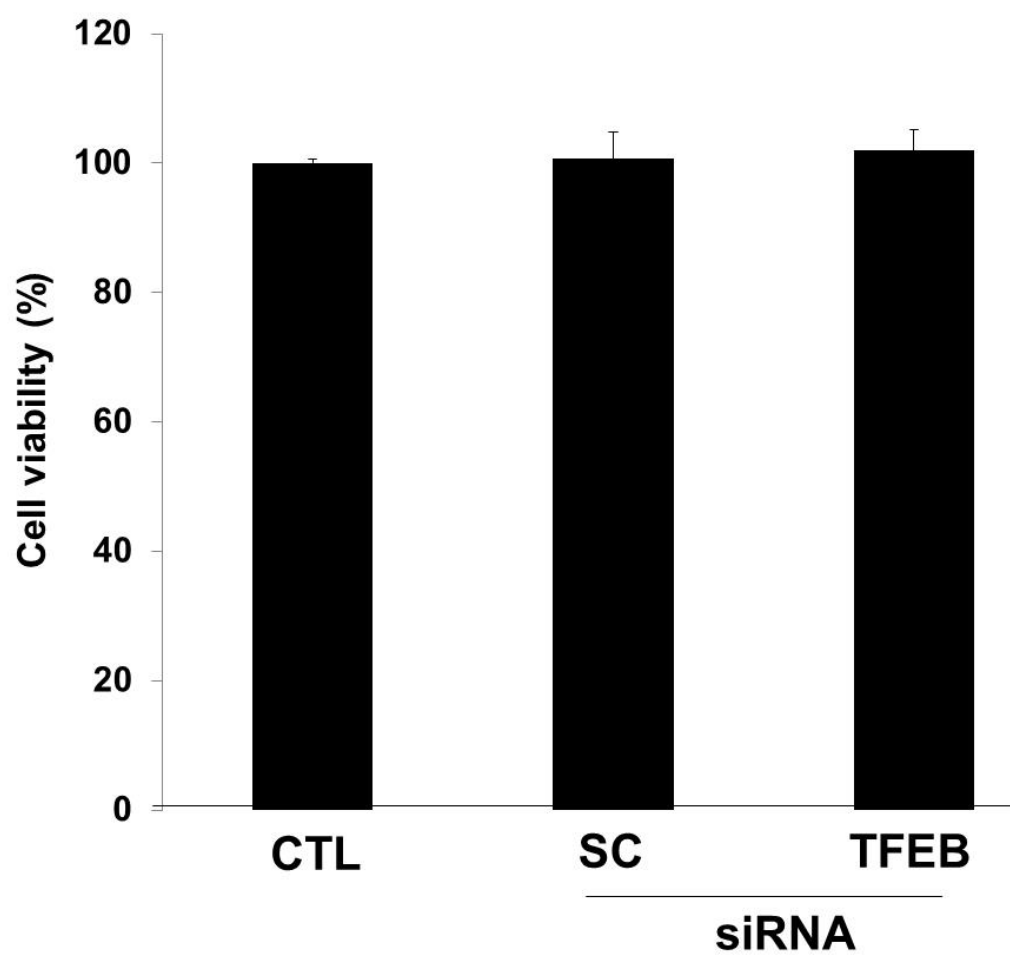

**Figure S1**

Supplement: FIG S1 [file sph002182506sf1.pdf]

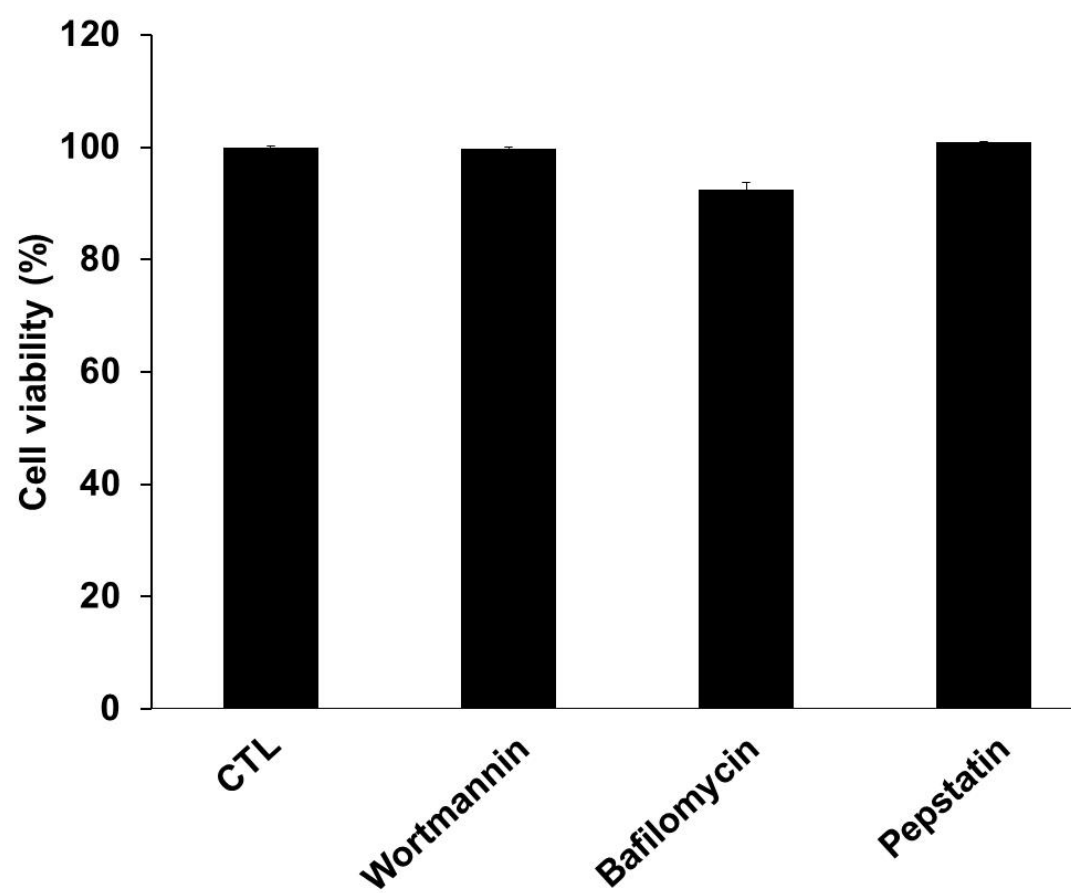

Figure S2

Supplement: FIG S2 [file sph002182506sf2.pdf]
